# Deathly triangle for pancreatic β-cells: Hippo pathway-MTORC1-autophagy

**DOI:** 10.1080/15548627.2021.1972404

**Published:** 2021-09-01

**Authors:** Amin Ardestani, Kathrin Maedler

**Affiliations:** a Centre for Biomolecular Interactions Bremen, University of Bremen, Bremen, Germany; b Department of Molecular Medicine, School of Advanced Technologies in Medicine, Tehran University of Medical Sciences, Tehran, Iran

**Keywords:** Autophagy, beta cells, diabetes, LATS2, MTORC1, type 2 diabetes

## Abstract

A progressive decline in the macroautophagic/autophagic flux is a hallmark of pancreatic β-cell failure in type 2 diabetes (T2D) but the responsible intrinsic factors and underlying molecular mechanisms are incompletely understood. A stress-sensitive multicomponent cellular loop of the Hippo pathway kinase LATS2 (large tumor suppressor 2), MTOR (mechanistic target of rapamycin kinase) complex 1 (MTORC1) and autophagy regulates β-cell survival and metabolic adaptation. Chronic metabolic stress leads to LATS2 hyperactivation which then induces MTORC1, subsequently impairing the cellular autophagic flux and consequently triggering β-cell death. Reciprocally, under physiological conditions, autophagy controls β-cell survival by lysosomal degradation of LATS2. These signaling cross-talks and the interaction between autophagy and LATS2 are important for the regulation of β-cell turnover and functional compensation under metabolic stress.

We are celebrating the 100th anniversary of the discovery of insulin by Frederick Banting and Charles Best. This historical milestone has enabled the treatment of diabetes and encouraged scientists to constantly move diabetes therapy forward. Despite this breakthrough and subsequent advances, there is still no causative therapy to prevent or slow down the loss of functional insulin-producing pancreatic β cells, a central hallmark of both type 1 diabetes and type 2 diabetes (T2D).

The highly conserved catabolic process macroautophagy (hereafter “autophagy”) is responsible for the turnover of long-lived proteins as well as clearance of aged or damaged organelles in order to maintain the physiological structure, quality and function of the cell. Vast research of the last 15 years indicates that autophagy is instrumental for various aspects of the life of β cells including insulin secretion, cellular homeostasis, metabolic compensation, stress response and survival. Highly regulated autophagy is critically required to protect β-cell mass and function, to empower their adaptive response to nutrient excess, high insulin demands or stress. Inhibition of autophagy emphasizes the fundamental importance of basal as well as stress-induced autophagy in β cells; perturbed autophagy compromises β-cell survival and compensatory expansion, thus ultimately leading to hyperglycemia and impaired glucose homeostasis.

Any impairment in the autophagic flux as “a measure of the autophagic degradation activity” is a key element of β-cell failure, shown in rodent models of diabetes as well as in individuals with T2D. This makes autophagy an essential process to preserve β-cell structure and function and a safeguard against diabetogenic conditions. Specific intrinsic factor(s) which regulate β-cells’ protective autophagy to cope against metabolic stress are not entirely known. In our recent study [1], we have identified a ubiquitously expressed serine/threonine kinase, LATS2 (large tumor suppressor 2), and core signaling component in the Hippo pathway, which constitutes a dynamic mutual interaction with autophagy in pancreatic β cells. LATS2 activity as well as its molecular partner MOB1 are highly induced in metabolically stressed β cells. Forced overexpression of LATS2 fosters human and mouse β-cell death and abolishes glucose-stimulated insulin secretion. Conversely, genetic inhibition of LATS2 or MOB1 maintains β-cell viability under pro-diabetic conditions placing LATS2 as an indispensable factor in β-cell apoptotic signaling. Also, selective disruption of *Lats2* in β cells *in vivo* restores normoglycemia, β-cell function and survival as well as the β-cell compensatory response in mouse models of diabetes.

How does LATS2 mechanistically control β-cell survival? We have determined that LATS2 activates MTOR (mechanistic target of rapamycin kinase) complex 1 (MTORC1), a well-established natural inhibitor of autophagy, through small RRAG GTPases (classical MTORC1 activators). This provides clarity on the recent MTORC1 paradox: widely known as a master regulator of cell growth and viability under physiological conditions, MTORC1’s constitutive inappropriate induction (“hyperactivation”) in diabetic β-cells leads to failure through diminished protective β-cell autophagy. Indeed, several recent studies have found sustained upregulation of MTORC1 activity in islets from patients with T2D and have thus linked MTORC1 hyperactivation with β-cell failure. We could show that inhibition of MTORC1 by genetic or pharmacological means restores β-cell survival in LATS2-overexpressing rodent and human islets/β cells suggesting that MTORC1 acts as a downstream signal in the LATS2-initiated apoptotic pathway.

Given that LATS2 induces MTORC1 and MTORC1 inhibits autophagy, we then tested whether LATS2 overexpression/deficiency affects β-cell apoptosis under conditions of diminished autophagy. Apoptosis induced by autophagy inhibitors or by knockdown of autophagy-related gene *Atg7* can be potentiated by LATS2 ectopic overexpression and blocked by LATS2 silencing, clearly underscoring balanced regulation of the LATS2-autophagy axis for β-cell survival. Also, β-cell apoptosis by LATS2 overexpression is accommodated with protein aggregate accumulation of autophagic substrates SQSTM1/p62 and LC3-II (the lipidated form of LC3 associated with autophagosomes), whereas LATS2 knockdown markedly restores autophagic flux. Similarly, pancreatic β cells from high fat-diet (HFD) fed diabetic mice reveal increased SQSTM1-positive β cells together with elevated levels of ribosomal protein RPS6 phosphorylation (p-RPS6; an MTORC1 readout), signs of deregulated MTORC1-autophagy and impairment of autophagic flux. Both are reversed in β-cell specific *lats2*-knockout mice, suggesting that autophagic flux is sufficiently enhanced to address the increased demand for proteolysis in the HFD setting. These data demonstrate that LATS2 restricts autophagic flux and is a key component of defective autophagy-induced apoptosis in β cells.

An intriguing observation for us was that endogenous LATS2 protein is elevated by autophagy inhibition, proposing LATS2 as a possible substrate for autophagy-mediated degradation. Such findings could be supported by the lysosomal localization of LATS2 through purification of intact lysosomes as well as its microscopy colocalization with LAMP1-containing compartments. Further, genetic silencing of autophagy machinery components, direct analysis of intact isolated autophagosomes and the LATS2-LC3 interaction confirms LATS2 as target of the autophagy machinery and identifies macroautophagy as the major autophagy pathway for degradation of LATS2 in β cells.

The finding that LATS2 operates both upstream and downstream of autophagy adds a further layer of complexity. We think that the degradation of LATS2 by autophagy conserves basal autophagy under the β-cell compensatory phase where “good” MTORC1 (MTORC1-mediated anabolic growth) is temporarily activated (likely by signals other than LATS2).

Collectively, under physiological or acute stress conditions, autophagy maintains β-cell survival by targeting LATS2 destruction as an endogenous adaptive mechanism through a negative-feedback loop. Under chronic metabolic stress (e.g., insulin resistance), LATS2 fosters activation of MTORC1, which then contributes to autophagy inhibition, subsequent accumulation of LATS2 and induction of apoptotic signaling (Figure 1). A vicious cycle starts from here, with diabetes at its end.

**Figure 1.**
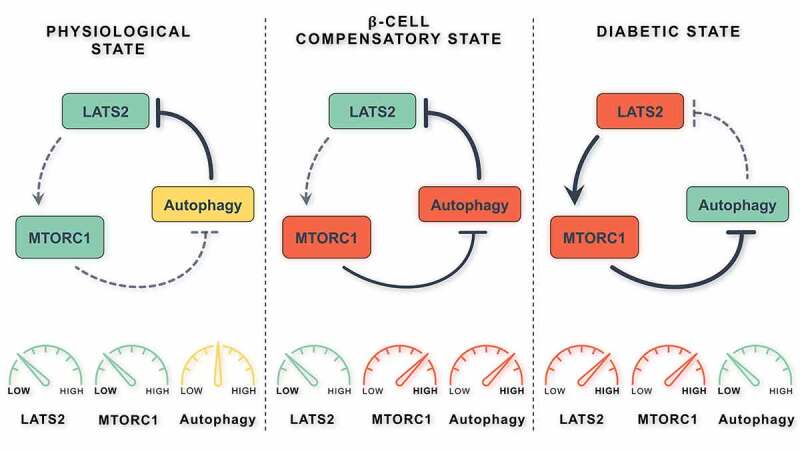
The LATS2-MTORC1-autophagy triangle in pancreatic β cells. Under physiological conditions, LATS2 is minimally active through autophagy-mediated lysosomal degradation. In the β-cell compensatory phase, when β-cell replication and enhanced insulin secretion are needed, MTORC1 is active to support such anabolic responses. In this case, MTORC1-directed inhibition of autophagy is important for the fine tuning and proper termination of autophagy. At the diabetic state, the association between LATS2-MTORC1 and autophagy leads to β-cell failure under a deteriorating metabolic situation. In this scenario, LATS2 is abnormally activated in response to nutrient excess and chronic metabolic stress, initiating sustained activation of MTORC1 followed by its deleterious actions. Consequently, stress-induced autophagy as an adaptive response to promote cell survival is compromised leading to LATS2 accumulation and amplification of apoptotic signaling in the β cells [1]

Uncovering the molecular links of LATS2-MTORC1-autophagy and β-cell failure is only at its beginning and further mechanistic characterization of their dynamic molecular communication is needed. Also, whether this LATS2-autophagy crosstalk extends beyond β-cells will provide further clues about the network of autophagy regulation in other metabolically active cells. In the future, the important function of LATS2 in its circular regulation of autophagy ending in β-cell failure makes it a promising target for β-cell-directed diabetes therapy.
